# Betulinic acid decreases lipid accumulation in adipogenesis-induced human mesenchymal stem cells with upregulation of *PGC-1α* and *UCP-1* and post-transcriptional downregulation of adiponectin and leptin secretion

**DOI:** 10.7717/peerj.12321

**Published:** 2021-10-14

**Authors:** Sasithon Senamontree, Thitiporn Lakthan, Pornsri Charoenpanich, Chanpen Chanchao, Adisri Charoenpanich

**Affiliations:** 1 Department of Biology, Faculty of Science, Silpakorn University, Nakhon Pathom, Thailand; 2 Department of Food Technology, Faculty of Engineering and Industrial Technology, Silpakorn University, Nakhon Pathom, Thailand; 3 Department of Biology, Faculty of Science, Chulalongkorn University, Bangkok, Thailand

**Keywords:** Human mesenchymal stem cell, Betulinic acid, Adipogenesis, Osteogenesis, Brown adipocyte, UCP-1

## Abstract

**Background:**

Controlling cellular functions, including stem cell growth and differentiation, can be the key for the treatment of metabolic disorders, such as type II diabetes mellitus (T2DM). Previously identified as peroxisome proliferator-activated receptor gamma (*PPARγ*) antagonist, betulinic acid (BA) may have the capability to control stem cell homeostasis, benefiting T2DM treatment. In this study, the effects of BA on osteogenesis and adipogenesis mechanisms of human mesenchymal stem cells (hMSCs) were investigated.

**Results:**

We observed that BA increased hMSC osteogenesis by enhancing the alkaline phosphatase activity, calcium deposition, and mRNA expressions of osteogenic markers, namely, runt-related transcription factor 2, osteocalcin, and osteopontin. In addition, BA decreased hMSC adipogenesis with the decrease in glycerol-3-phosphate dehydrogenase activity, reduced intracellular lipid accumulations, down-regulated CCAAT-enhancer-binding protein alpha, and suppressed post-transcriptional adiponectin and leptin secretion. BA increased the brown adipocyte characteristics with the increase in the ratio of small lipid droplets and glucose uptake. Furthermore, the mRNA expressions of brown adipocyte markers, namely, *PPARγ* coactivator one alpha, uncoupling protein 1, and interleukin-6 increased.

**Conclusions:**

Our results uncovered the mechanisms of how BA improved glucose and lipid metabolisms by decreasing white adipogenesis and increasing brown adipogenesis. Altogether, BA may be used for balancing glucose metabolisms without the potential side effects on bone loss or weight gain.

## Introduction

Human mesenchymal stem cells (hMSCs) are progenitor cells of mesenchymal lineage tissues, including muscle, cartilage, bone, and fat. Within these tissues, several of the hMSCs are left as undifferentiated adult stem cells. These undifferentiated hMSCs in adults maintain their crucial roles in balancing tissue homeostasis and controlling their surrounding microenvironments (Chamberlain et al., 2007; Charoenpanich et al., 2011; Samuel et al., 2019). In particular, bone and adipose tissues are dynamic tissues that can be dramatically altered by physical activity, lifestyle behavior, and diet. The overexpansion of adipose tissue in obesity has an inverse effect on bone metabolisms by decreasing bone formation through the competitive utilization of the same mesenchymal progenitor cells and increasing bone resorption by inducing osteoclast activation with pro-inflammatory cytokine secretion (Abdallah, 2017; Cao, 2011; James, 2013; Lacey et al., 2009; Sonowal et al., 2013). Furthermore, the crosstalk between osteogenesis and adipogenesis can complicate metabolic disorders, such as diabetes mellitus, as evidenced in the case of type II diabetes mellitus (T2DM) treatments (Grey, 2009; Sardone et al., 2011). Further, patients with type I and T2DM also display the risk of osteoporotic fracture (Sellmeyer et al., 2016).

Betulinic acid (BA) is a naturally occurring pentacyclic triterpenoid with potential anti-obesity, anti-diabetes, anti-inflammation, and anti-osteoporosis properties (Kim et al., 2019; Ou et al., 2019; Park et al., 2014; Song et al., 2021). The treatment with BA at 10 and 30 mg/kg can restore the imbalance of inflammatory mediators (tumor necrosis factor alpha (TNF-α), interleukin 6 (IL-6), and IL-10) and prevent cecal ligation and puncture-induced kidney damage in mice (Lingaraju et al., 2015). The anti-inflammatory activity of BA through modulating TNF-α production by macrophages with IL-10-dependent mechanism was also reported in a lethal dose of lipopolysaccharide-treated BALB/c mice (Costa et al., 2014). Another anti-inflammation signaling pathway triggered by BA is through the activation of peroxisome proliferator-activated receptor gamma (PPARγ) as shown in the IL-1β-induced inflammation of osteoarthritis chondrocytes (Jingbo et al., 2015). PPARγ is the master regulator of adipogenesis and the target of insulin-sensitizing drug, thiazolidinediones (TZDs) (Kubota et al., 2006; Lehmann et al., 1995; Murakami-Nishida et al., 2019).

An *in vivo* study of mice fed with a high-fat diet (HFD) showed that BA prevented abdominal fat accumulation with the decrease in blood glucose, plasma triglycerides, and total cholesterol (de Melo et al., 2009). A study on African medicinal plant extract, *Diospyros bipindensis*, showed that BA had a high affinity to PPARγ and acted as PPARγ antagonist, which induced glucose uptake, inhibited adipogenesis in murine pre-adipocyte (3T3-L1 cells), and enhanced osteogenesis in murine pre-osteoblast (MC3T3-E1) (Brusotti et al., 2017). An *in vitro* study of MC3T3-E1 cells showed that BA enhanced osteogenesis through bone morphogenetic protein(BMP)/runt-related transcription factor 2 (RUNX2) and β-catenin signals (Lo et al., 2010). We hypothesized that BA can be a potent agent that triggers the balance of bone and adipose tissue formation in progenitor cells. Here, we addressed the mechanisms of BA triggering the balance between osteogenesis and adipogenesis in hMSCs. We reported that BA can increase hMSC osteogenesis and suppress hMSC adipogenesis. Additionally, we proposed that the suppression of adipogenesis by BA with increases in metabolic activity and glucose uptake might be through inducing brown fat formation in hMSCs.

## Materials & methods

### Cell culture

hMSCs were obtained from the Japanese Collection of Research Bioresources Cell Bank (JCRB Cell Bank, UE7T-13, JCRB1154, Lot #07072007). This hMSC cell line had been established for its multipotent differentiation, including osteogenesis and adipogenesis (Tsujimura, Kusamori & Nishikawa, 2019; Zhang et al., 2019). For cell proliferation, hMSCs were cultured in complete growth media (CGM: α-Minimum Essential Medium with 10% fetal bovine serum, 100 units/ml Penicillin-Streptomycin, and two mM L-glutamine) at 37 °C and 5% CO_2_. The medium was changed every 3 days, and the cells were sub-cultured after about 80% confluency. For cell differentiation, hMSCs were seeded in multi-well plates. When 80% confluency was reached (within 2–3 days), the CGM was replaced with osteogenic medium (OM) or adipogenic medium (AM). BA was purchased from Sigma-Aldrich (cat#B8936) and initially dissolved in 50 mM dimethyl sulfoxide (DMSO). The final concentrations of DMSO were kept below 0.05% in all conditions.

### Cell differentiation media

In this study, an OM recipe without dexamethasone was used to highlight the effect of BA. The OM is a CGM-based medium supplemented with 50 µM ascorbic acid and five mM β-glycerophosphate (Choi et al., 2016).

AM is also CGM-based with one µM dexamethasone, 10 µg/ml h-insulin, 100 µM indomethacin, and 500 µM isobutymethyxanthine (Nöth et al., 2002).

### Metabolic activity of hMSCs

alamarBlue™ dye (Invitrogen) is a resazurin-based assay that can be used for continuous monitoring of mitochondrial function, and it was performed for the metabolic activity analysis of hMSCs as per manufacturer’s instructions. For metabolic tracking, the cells were seeded at 5 × 10^3^ cells/well in 96-well plates and treated with BA at 5, 10, 15, and 20 µM for 7 and 10 days in CGM, OM, or AM. At the designated time points, the medium was replaced with fresh CGM with 10% alamarBlue and then incubated for 2 h before absorbance reading at 570 and 600 nm. The percentage of metabolic activity was compared with the control group without BA at the selected time points for each media type (Maxson & Burg, 2008; Quent et al., 2010).

### Evaluations of osteogenesis

The effects of BA on hMSC osteogenesis were evaluated in two levels, namely, alkaline phosphatase (ALP) enzymatic activity and bone matrix calcium deposition.

ALP is a critical enzyme in bone mineralization and known as an early marker of osteogenesis (Mohamadnia et al., 2007). For ALP activity assay, hMSCs were seeded at 5 × 10^3^ cells/well in 96-well plates and cultured in OM for 7 days. The ALP activity assay was performed as previously described (Montazerolghaem et al., 2016). Briefly, the cells were lysed in a lysis buffer, followed by three freeze-thawing cycles to fully break the cells. A total of one mg/ml p-nitrophenyl phosphate substrate in diethanolamine was added to the cell lysis at the ratio of 2:1. The reactions were incubated at 37 °C, and the absorbance was read at 405 nm every 5 min for 30 min. The total protein was measured with bicinchoninic acid assay (Walker, 1996). The enzymatic activity was calculated as described in Enzymatic Assay of Alkaline Phosphatase, Diethanolamine Assay (EC 3. 1. 3. 1):

${\rm Unit}/{\rm mg\; protein} = \displaystyle{{{\rm Unit}/{\rm ml\; enzyme}} \over {{\rm mg\; protein}/{\rm ml\; enzyme}}}$

Calcium deposition was quantified with calcium colorimetric assay with o-cresolphthalein, which was adapted from the work of Tu et al. (2014). Cells were seeded at 2 × 10^4^ cells/well in 24-well plates and cultured in OM with 5, 10, 15, and 20 µM BA or control without BA in OM and CGM. After 14 days, the cells were washed twice with phosphate-buffered saline (PBS). Then, the accreted calcium was dissolved in 0.1 M HCl, shaken at 4 °C for 8 h, and centrifuged at 500×g for 2 min. The supernatant with dissolved calcium was then used for calcium quantification. A chromogenic reagent 190 µl (1.1 mM O-cresolphthalein complexone and 7.9 mM 8-hydroxyquinoline) was added to the 10 µl sample, and the absorbance was read at 570 nm.

To visualize the calcium nodule, we washed the cells twice with PBS, followed by 4% paraformaldehyde fixation for 10 min and staining with 2% alizarin red.

### Evaluations of adipogenesis

The adipogenesis of hMSCs was analyzed with glycerol 3-phosphate dehydrogenase (GPDH) activity and intracellular lipid droplet accumulation.

Cell culture and cell lysate preparation for GPDH activity assay were performed with the same protocol as the ALP activity, but with the AM used for cell culture instead of an OM. Exactly 90 µl 334 µM b-nicotinamide adenine dinucleotide, which is the reduced form of NADH, was added to 100 µl cell lysate and then further incubated at 37 °C for 10 min before adding 10 µl four mM dihydroxyacetone phosphate. The absorbance was read at 340 nm every 5 min for 30 min. GPDH was calculated and normalized with the total protein (Sottile & Seuwen, 2001).

Oil Red O and Nile red staining were used to analyze the intracellular lipid accumulation. Oil Red O is a common oil droplet stain that can also be used to interpret the total triglyceride content by dissolving the dye for absorbance reading at 540 nm (Kanawa et al., 2013). hMSCs were cultured in an AM in 96-well plates for 21 days. Then, the cells were washed twice with PBS, followed by 4% paraformaldehyde fixation for 10 min. The oil droplets were stained with Oil Red O (1.8 mg/ml in 60% isopropanol) for 10 min. The excess dye was then removed by washing with distilled water. The stained lipid was then dissolved in 100 µl isopropanol and read at 540 nm.

For the analysis of lipid droplet size and number, the cells were cultured in 48-well plates containing the AM for 21 days and then stained with 10 µg/ml Nile red (1% acetone in non-phenol red Dulbecco’s Modified Eagle Medium) (Hogan et al., 2016). Images were captured with a fluorescence microscope (Olympus, CKX53) with the excitation wavelength at 495 nm, with five images obtained per well in triplicate and analyzed with ImageJ using function analyze the particles (Deutsch et al., 2014). Briefly, the images were binarized to black and white pixels. Then, watershedding techniques were applied to measure individual lipid droplets by size and perimeter.

### Glucose uptake

The glucose uptake by hMSCs during adipogenesis was analyzed on days 10 and 14. Cells were seeded in 48-well plates at 1 × 10^4^ cell/well. After 10 or 14 days, the medium was replaced with a fresh medium, and the glucose level was measured with Accu-chek guide (Kim et al., 2018) after 4 and 8 h.

### Cellular mechanisms

mRNA gene expression analysis was performed with real-time reverse-transcription polymerase chain reaction (qRT-PCR). hMSCs were seeded in 24-well plates at 2 × 10^4^ cell/well. The total RNA was extracted with Total RNA Mini Kit (Favorgen, FATRK001) and converted to cDNA with HyperScript™ RT master mix with oligo dT (GeneAll, 601–703). The quantitative PCR was performed with the Applied Biosystems™ 7,500 Real-Time PCR System using iTaq Universal SYBR Green Supermix (Biorad, 172–5,122). The PCR products were checked with melting temperature analysis. The ΔCT was calculated using GAPDH. The relative gene expressions were calculated using the 2^−ΔΔCT^ method with the cut-off at twofold change and *P* < 0.05 (Rao et al., 2013). Table 1 shows the primer sequences.

**Table 1:** Primer sequences for qRT-PCR.

Gene	Forward (5–3′)	Reverse (5–3′)	Product length
Adiponectin	TCCTGCCAGTAACAGGGAAG	GGTTGGCGATTACCCGTTTG	89
*ALP*	CCCAAAGGCTTCTTCTTG	CTGGTAGTTGTTGTGAGC	357
β-catenin	GATTTGATGGAGTTGGACATGG	TGTTCTTGAGTGAAGGACTGAG	218
*BMP2*	ATGGATTCGTGGTGGAAGTG	GTGGAGTTCAGATGATCAGC	349
*BMP7*	GGTCATGAGCTTCGTCAACC	GCAGGAAGAGATCCGATTCC	236
*C/EBP-α*	AAGAAGTCGGTGGACAAGAACAG	TGCGCACCGCGATGT	70
*FABP4*	GCTTTGCCACCAGGAAAGTG	ATGGACGCATTCCACCACCA	281
*GAPDH*	GTTCCAATATGATTCCACCC	AGGGATGATGTTCTGGAGAG	487
*GLUT-4*	CTTCGAGACAGCAGGGGTAG	ACAGTCATCAGGATGGCACA	168
*IL-6*	GGTACATCCTCGACGGCATCT	GTGCCTCTTTGCTGCTTTCAC	81
Leptin	TCCCCTCTTGACCCATCTC	GGGAACCTTGTTCTGGTCAT	110
*LEP-R*	GCTATTTTGGGAAGATGT	TGCCTGGGCCTCTATCTC	499
*MYF5*	TTCTACGACGGCTCCTGCATA	CCACTCGCGGCACAAACT	67
*OC*	GGCGCTACCTGTATCAATGG	TCAGCCAACTCGTCACAGTC	106
*OPN*	CATGAGAATTGCAGTGATTTGCT	CTTGGAAGGGTCTGTGGGG	186
*PGC-1α*	GTTCCCGATCACCATATTCCA	GCGGTGTCTGTAGTGGCTTGA	101
*PPARγ-2*	CAGTGTGAATTACAGCAAACC	ACAGTGTATCAGTGAAGGAAT	101
*PRDM16*	CAGCACGGTGAAGCCATTC	GCGTGCATCCGCTTGTG	87
*RUNX2*	GTACAGCTTTAAGGATTCCCTCAATTC	TTGCTAATGCTTCGTGTTTCCA	86
*TNF- α*	CAGAGGGAAGAGTTCCCCAG	CCTTGGTCTGGTAGGAGACG	325
*UCP-1*	CTGGAATAGCGGCGTGCTT	AATAACACTGGACGTCGGGC	101
*WNT3A*	ACTACGTGGAGATCATGCCC	ATGAGCGTGTCACTGCAAAG	210

The release of adipokines, adiponectin, IL-6, leptin, and TNF-α was measured with Milliplex Map Human Adipokine Magnetic Bead Panel Kit. hMSCs were seeded at 10^4^ cells/well in 48-well plates. The culture medium was changed by half every 3 days and collected for cell-secreted adipokines on days 10, 14, and 17. The detection and analysis were performed in accordance with the manufacturer’s instructions. At least 50 beads were counted for each replicate, and the results were calculated as pg/ml by comparison with the manufacturer’s standard control (Chen et al., 2014).

### Statistical analysis

All statistical analysis was performed with 27^th^ edition of SPSS. Data are shown as mean ± standard deviation from three biological replicates. The Shapiro–Wilk test was applied to assess normality of the distributions. In cases of a normal distribution, one-way ANOVA with Tukey multiple comparison for equal variances or Dunnett’s C multiple comparison for unequal variances were used. In cases of a non-normal distribution, the Kruskal–Wallis test with Mann Whitney U test was used to determine the level of significance between the groups. *P* values < 0.05 were accepted as significant.

## Results

### BA increased the metabolic activity of hMSCs during adipogenesis

**Figure 1:**
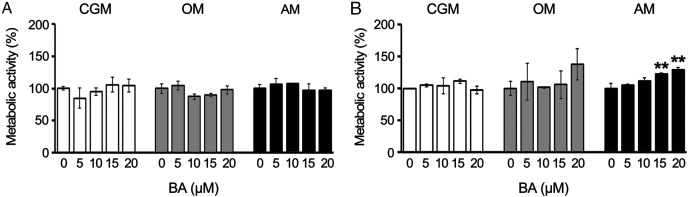
BA increased the metabolic activity of hMSCs during adipogenic differentiation. The metabolic activity of hMSCs was tracked with alamarBlue assay during the proliferating phase (CGM), osteogenic differentiation (OM), and adipogenic differentiation (AM) on days 7 (A) and 10 (B). **P < 0.01. Error bars represent standard deviation (*n* = 3).

To track the changes in the metabolic activity of hMSCs in response to BA, we performed alamarBlue assays on days 7 and 10, proliferating phase, osteogenic differentiation, or adipogenic differentiation. The results showed that BA caused no alteration in the metabolic activity of hMSCs during the proliferating phase or osteogenic differentiation. The BA at 15, and 20 µM increased the metabolic activity of hMSCs during adipogenesis by 22% and 29%, respectively, on day 10 (Figs. 1A and 1B) without changes in total protein content (Fig. S1).

### BA increased osteogenic differentiation and decreased adipogenic differentiation of hMSCs, changing calcium deposition, lipid accumulation, enzyme activities, and gene expressions

**Figure 2:**
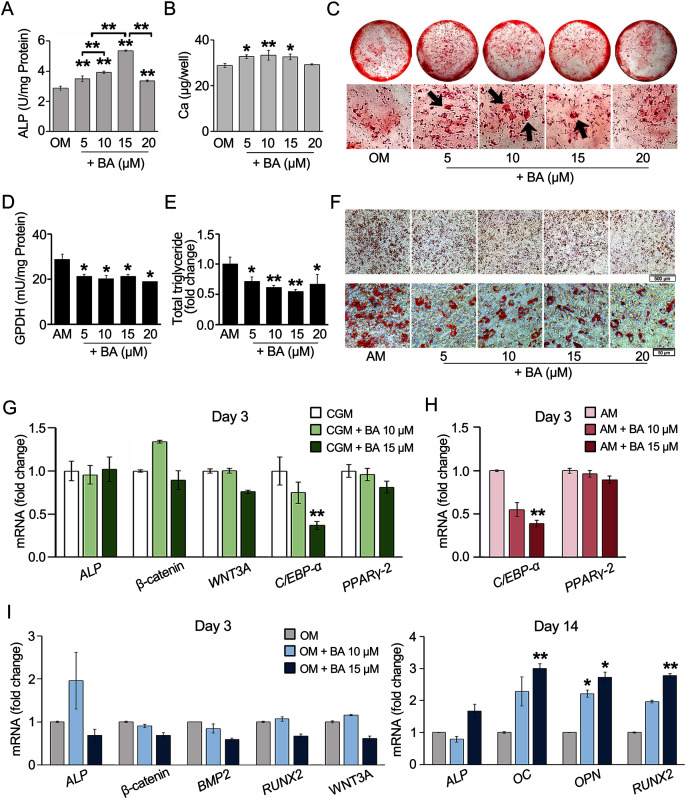
BA increased hMSC osteogenic differentiation but decreased hMSC adipogenic differentiation. For osteogenesis, ALP activity was analyzed on day 7 (A), whereas calcium deposition was assessed on day 14. Calcium was quantified with calcium colorimetric assays (B) and visualized for calcium nodule (arrows) with alizarin red staining (C). For adipogenesis, GPDH activity was analyzed on day 7 (D), whereas lipid droplet formation was stained with Oil Red O (F) and dissolved for quantification on day 21 (E). qRT-PCR analysis of hMSCs showed the effect of BA on the mRNA expressions of osteogenic and adipogenic markers during proliferating phase (G), adipogenic markers during adipogenesis (H), and osteogenic markers during osteogenesis (I). A, B, D, E *P < 0.05 and **P < 0.01. G, H, I statistical difference at *P < 0.05, **P < 0.01 and at least 2-fold changes.

The effects of BA on hMSC osteogenesis or adipogenesis were investigated with enzyme activity assays, calcium deposition, or lipid accumulation. The results showed that ALP, the key enzyme in bone mineralization, significantly increased in hMSCs with a dose-dependent response to BA at 5–15 µM (Fig. 2A). At 15 µM, the ALP activity increased the most by 85%. Calcium accretion was also significantly increased by BA at 5, 10, and 15 µM as shown in the calcium quantification assay, with the average increase of about 4–5 µg/well or 15%–18% (Fig. 2B). With alizarin red staining, thicker and larger calcium nodules were observed in BA treatments at 5, 10, and 15 µM (Fig. 2C).

For hMSC adipogenesis, GPDH, the key enzyme in lipid biosynthesis, and its intracytoplasmical accumulation lipid were investigated. Our preliminary result showed elevated activity of GPDH in hMSCs by adipogenic induction at day 7 but not day 14 (Table S1). Day 7 was then selected as the timepoint for GPDH activity analysis. The results showed that BA at 5–20 µM significantly decreased the GPDH activity by 26%–34% (Fig. 2D). hMSC adipogenic differentiation was confirmed with Oil Red O staining (Fig. S2). At day 21, Oil Red O staining showed evident reduction in lipid accumulation with the decrease in total lipid content by 28%–45% (Figs. 2E and 2F).

BA can alter the hMSC lineage differentiations by enhancing bone formation with the increase in ALP enzymatic activity and bone matrix deposition and reducing fat cell formation with the demolishment of lipid droplet accumulation and GPDH enzyme activity.

Further, hMSC gene expression was analyzed with qRT-PCR. The effects of BA without additional differentiation supplements in the CGM were monitored on day 3. We observed that the effects of BA on hMSC early osteogenic gene expressions, namely *ALP*, β-catenin, and Wnt family member 3A (*WNT3A*) were not evident without osteogenic induction factors (Fig. 2G). However, the expression of adipogenic marker CCAAT-enhancer-binding proteins alpha (*C/EBP-α*) level was significantly reduced by BA alone (Fig. 2G). In addition, the remarkable downregulation of *C/EBP-α*, but not *PPARγ-2*, by BA was observed in hMSCs during adipogenic induction (Fig. 2H).

With osteogenic induction factors, BA significantly upregulated the expressions of late osteogenic markers, *ALP*, osteocalcin (*OC*), osteopontin (*OPN*), and *RUNX2* on day 14 (Fig. 2I). However, the expression of early osteogenic marker genes, *i.e*., *ALP*, β-catenin, bone morphogenetic protein 2 (*BMP2*), *RUNX2*, and *WNT3A*, was not altered (Fig. 2I).

### BA down-regulated *C/EBP-α*, upregulated *IL-6* mRNA expression, but post- transcriptionally down-regulated adiponectin and leptin secretion in hMSCs

**Figure 3:**
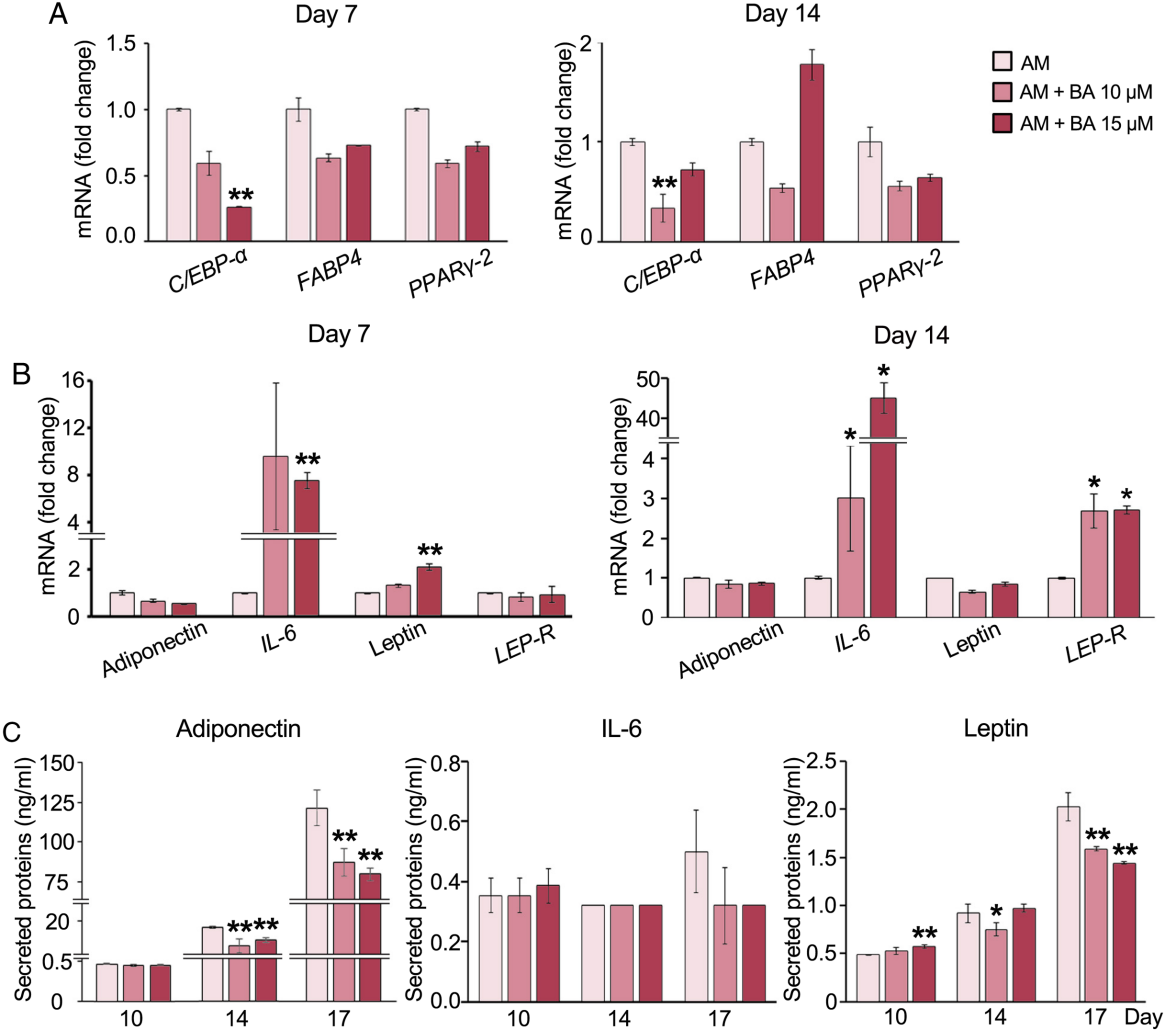
Effect of BA on white adipogenic markers and adipokine expressions on hMSCs. mRNA expressions of white adipocyte markers (A) and adipokines (B) were measured by qRT-PCR on days 7 and 14. The secreted adiponectin, IL-6, and leptin were measured with Milliplex Map Human Adipokine Magnetic Bead Panel Kits (C). Results are displayed as mean ± standard deviation (*n* = 3). *P < 0.05 and **P < 0.01.

The decreased adipogenesis of hMSCs by BA with the downregulation of *C/EBP-α* expressions was remarkable at all time points on days 3, 7, and 14 (Figs. 2H and 3A), whereas the expressions of *PPARγ-2* and fatty acid-binding protein 4 (*FABP4*) were not significantly altered (Fig. 3A).

Further, adipokine mRNA expressions were analyzed with qRT-PCR. The result showed that BA at 15 µM significantly increased the expression of *IL-6* and leptin on day 7 (Fig. 3B). Meanwhile, the mRNA expressions of adiponectin and leptin-receptor (*LEP-R*), were not altered by BA on day 7 (Fig. 3B). On day 14, both *IL-6* and *LEP-R* expressions were significantly increased by BA at 10 and 15 µM, while the expressions of adiponectin and leptin were not altered (Fig. 3B).

Further, the secreted adipokines were analyzed with Milliplex Map Human Adipokine Magnetic Bead Panel Kits. The pilot results comparing the adipokines secreted by hMSCs during adipogenic and proliferating phases showed that the secretions of adiponectin and leptin were induced tremendously after 14 days in adipogenic induction media by about 1,400-and 11-fold, respectively (Table 2). On the contrary, the IL-6 secretion of hMSCs extensively decreased with adipogenic induction by more than 100-fold. TNF-α secretion was minimal during hMSC proliferation in the lower range of less than two pg/ml and diminished during adipogenesis (Table 2). We then analyzed the effect of BA during adipogenesis on hMSC-secreted adiponectin, IL-6, and leptin. The secreted adiponectin and leptin significantly decreased on days 14 and 17 with BA treatment (Fig. 3C). The secreted IL-6 was not altered by the addition of BA during adipogenesis (Fig. 3C).

**Table 2:** Adipokine secretions by hMSCs at day 14 showed the basal protein expressions of adiponectin, IL-6, leptin, and TNF-α (CGM) and their changes during adipogenic induction (AM).

Adipokine	Level of adipokine secretion	
	CGM (pg/ml)	AM (pg/ml)
Adiponectin	<13.91	19549.00
IL-6	340.15	0.32
Leptin	81.82	969.95
TNF-α	1.58	<0.40

### BA potentially shifted adipogenic lineage differentiation of hMSCs toward brown adipocytes with increased glucose uptake and enhancement of *PPARγ* coactivator one alpha (*PGC-1α*) and uncoupling protein 1 (*UCP-1*) expressions

Recent studies showed that hMSCs can give rise to the distinctive lineages of white or brown adipocytes (Park, Kim & Bae, 2014). To determine the effect of BA on brown adipocyte formation, we analyzed lipid droplet formation and glucose uptake along with the mRNA expression of brown adipocyte markers.

**Figure 4:**
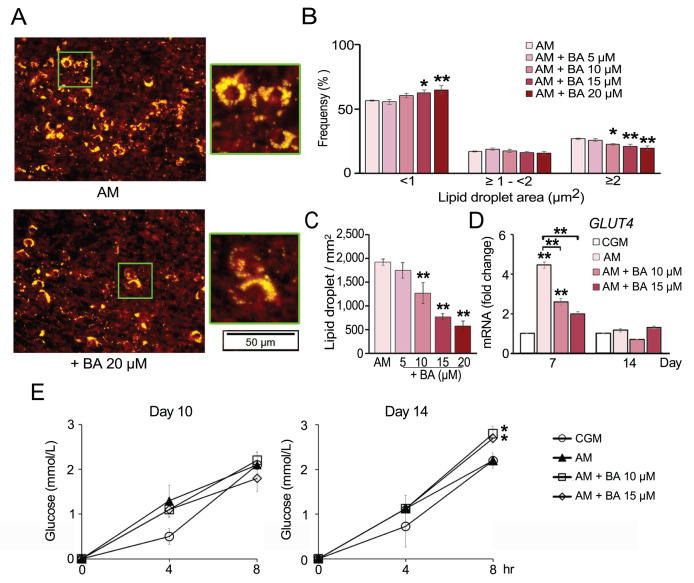
Effect of BA on hMSC lipid droplet formation and glucose uptake. Nile red staining showed reduction in the number (A and C) and size of lipid droplets (A and B). The mRNA expression of glucose transporter 4 (*GLUT4*) was quantified on days 7 and 14 (D). Glucose uptake of hMSCs was analyzed on days 10 and 14 (E). Results are displayed as mean ± standard deviation (*n* = 3). *P < 0.05 and **P < 0.01.

The number of lipid droplets stained by Nile red were aligned with the results from Oil Red O staining. BA significantly reduced the amount of lipid droplets (Figs. 4A and 4C). Moreover, lipid droplet size distribution analysis revealed that BA decreased the ratio of large lipid droplets (>3 µm^2^) and increased the ratio of small-sized lipid droplets (<1 µm^2^) (Fig. 4B). A small lipid droplet size is one of the characters that can be used to distinguish brown adipocytes from white adipocytes (Barneda et al., 2013).

Glucose uptake by hMSCs was analyzed on days 10 and 14. The results showed that BA did not alter glucose uptake during adipogenesis of hMSCs on day 10 but significantly increased the glucose uptake on day 14 (Fig. 4E). Glucose transporter 4 (*GLUT4*) mRNA expression was quantified on days 7 and 14. The results showed that *GLUT4* expression was significantly increased by adipogenic induction on day 7 and decreased with BA treatment, while there was no change in GLUT4 expression on day 14 (Fig. 4D).

**Figure 5:**
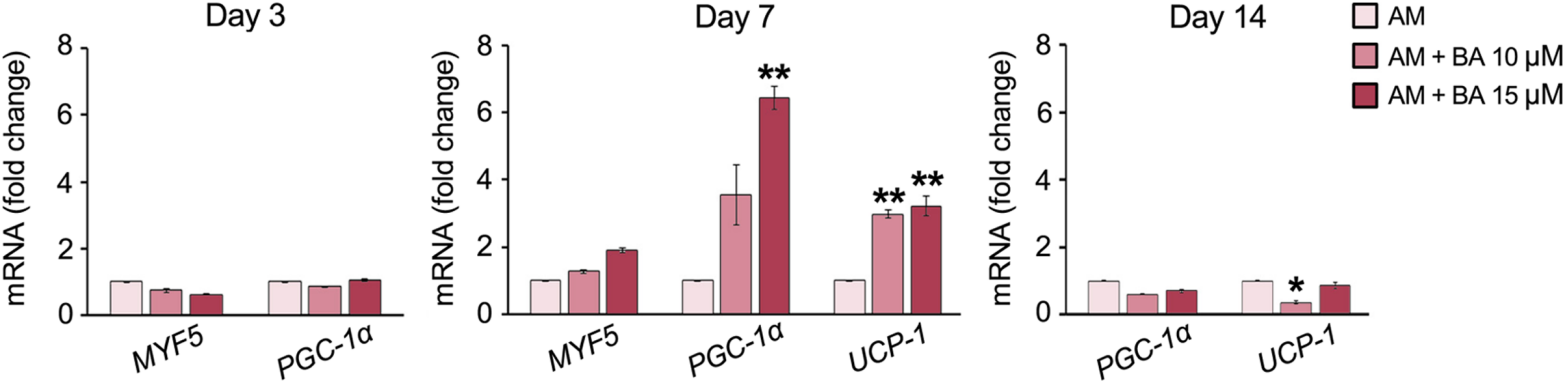
BA increased the expressions of brown adipogenic markers, *PGC-1α*, and *UCP-1* on day 7 of hMSC adipogenesis. Results are displayed as mean ± standard deviation (*n* = 3). *P < 0.05 and **P < 0.01.

The expressions of five brown adipocyte markers, namely, bone morphogenetic protein 7 (*BMP7*), transcriptional co-regulator PR domain-containing 16 (*PRDM16*), myogenic factor 5 (*MYF5*), *PGC-1α*, and *UCP-1* were analyzed. The basal expression of maker genes during adipogenesis on day 7 showed that this hMSC cell line, UE7T-13, did not express *BMP7* nor *PRDM16* with a cycle threshold (Ct) over 35 (data not shown). Meanwhile, *MYF5*, *PGC-1α*, and *UCP-1* were normally expressed in hMSCs, BA enhanced the expressions of *PGC-1α* and *UCP-1* on day 7 by up to 6.42 and 3.22-fold, respectively (Fig. 5). On day 14, the expression of *PGC-1α* was not affected by BA, and the expression of *UCP-1* was decreased by BA at 10 µM.

## Discussion

**Figure 6:**
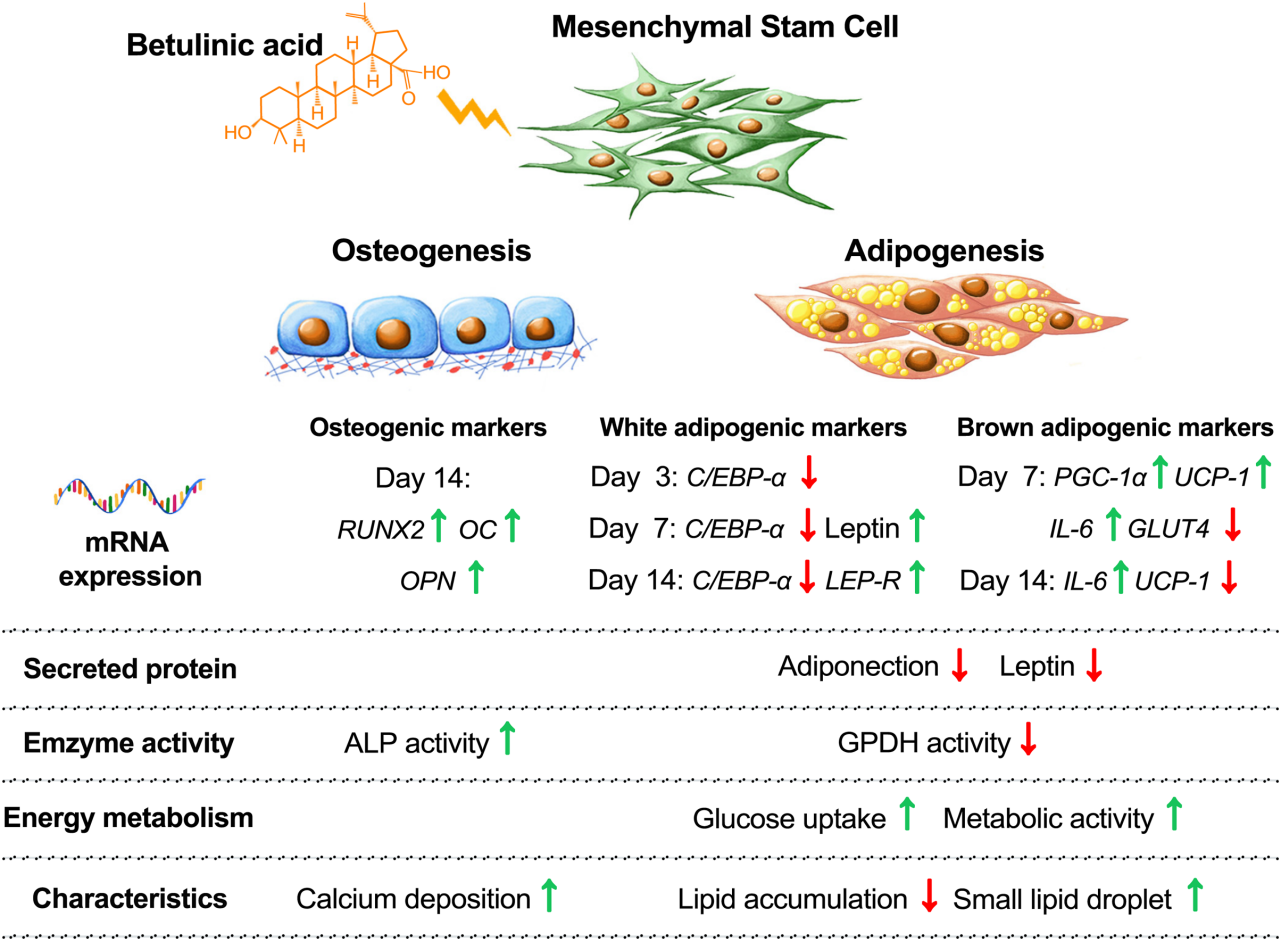
Concluding schematic shows how BA alters the fate of hMSC toward osteogenesis and brown adipogenesis and away from white adipogenesis.

Balancing cellular metabolisms by altering lipid synthesis and storage are intricate with diabetes causes and treatments. Cellular glucose uptake, lipid metabolism, adipose tissue formation, and energy utilization all play parts in balancing the blood glucose level. In addition, increasing the risk of osteoporosis and bone fractures have been associated with diabetes (de Paula, Horowitz & Rosen, 2010). In this study, we observed that BA attuned hMSC differentiation by increasing osteogenesis, decreasing white adipogenesis, and increasing brown adipogenesis with the upregulation of *UCP-1*, *PGC-1*α, and *IL-6* mRNA expressions, and post-transcriptional downregulation of adiponectin and leptin secretions (Fig. 6).

Hyperglycemia has direct and in-direct effects on bone formation and resorption by suppressing osteoblast cell growth and mineralization, increasing the number of osteoclasts, upregulating their markers, and inducing lipid formation within the bone marrow (Wongdee & Charoenphandhu, 2011). Increased risk of bone fracture was also reported when using the anti-diabetic drug, TZDs (Chen et al., 2015; Schwartz & Sellmeyer, 2007). Acting as the PPARγ agonist, this drug directly suppresses osteoblast development and induces bone loss (Baroi et al., 2021; Jackuliak, Kužma & Payer, 2019; Takada et al., 2007). On the contrary, the downregulation of the prime osteogenic regulator, *Runx2*, is also required for adipogenesis. The overexpression of *Runx2* can inhibit pre-adipocyte adipogenic differentiation and its trans-differentiation to bone cells (Zhang et al., 2012). In this study, we observed that BA increased hMSC osteogenesis by increasing the expression of *RUNX2*, *OC*, and *OPN* but did not directly alter the mRNA expression of *PPARγ* during adipogenesis. A previous study showed that BA can act as PPARγ antagonist by binding with PPAR receptors without transactivation activities (Brusotti et al., 2017).

Studies on synthetic PPAR antagonist showed that blocking or reducing the activity of PPARγ can reduce HFD-induced adipocyte hypertrophy and insulin resistance by reducing the size of adipocytes and decreasing the secretion of TNF-α and leptin (Abbas et al., 2013; Rieusset et al., 2002). However, the level of TNF-α secreted by hMSCs during adipogenesis in this study was below the detection limit. Thus, the effect of BA cannot be measured. The remarkable decrease in leptin secretion on day 17 by BA in hMSCs (Fig. 3C) without the decrease in leptin mRNA expression (Fig. 3B) suggested the effect of BA on secreted leptin at the levels of translation or secretion. Previous study on insulin treatment of adipose tissues from fed or starved rats also showed the changes in leptin secretion by altering leptin translation without alteration in leptin mRNA expression (Lee et al., 2007). Also, a decrease in leptin secretion in human adipose tissue by adrenergic activation was triggered at the level of leptin releasing from the preformed pool (Ricci et al., 2005).

BA has been extensively studied for its anti-cancer and anti-viral activities (Moghaddam, Ahmad & Samzadeh-Kermani, 2012). Recent studies showed its potential roles in balancing immunity and metabolisms including bone and lipid metabolisms (Birgani et al., 2018; Mohsen et al., 2019; Ríos & Máñez, 2018). It has been shown that BA reduced adipogenesis in mouse preadipocytes with decreases in *C/EBP-α, PPARγ*, *GLUT4*, cation diffusion facilitator, and perilipin one expressions (Brusotti et al., 2017). Also, BA increased glucose uptake rate in differentiated mouse adipocyte both with and without insulin stimulation (Brusotti et al., 2017). In this study, we also found that BA decreased the expressions of *C/EBP-α* and *GLUT4* while increased metabolic activity and glucose uptake in hMSCs during induced adipogenesis. After 11 weeks of BA treatment in HFD mice, BA helped maintain body temperature during acute cold exposure, decreased body weight, while increased brown fat tissues (Kim et al., 2019). Study of glucose overload in rat *in vivo* showed that BA helped maintain serum glucose level, increased serum insulin level, and increased muscle glycogen content (Castro et al., 2014). Further, *in vitro* study with dissected muscles showed that BA increased glucose uptake *via* increasing translocation of GLUT4 to plasma membrane (Castro et al., 2014). With BA increased metabolic activity and glucose uptake of hMSCs in our study and the new finding on BA improving energy balance with increase glucose uptake, and BAT formation in HFD mice (Kim et al., 2019), we then focused on the possibility of BA driving hMSCs toward brown adipocytes rather than just suppressing white adipocyte formation.

The formation of white and brown adipocytes requires the regulation by PPARγ and C/EBP-α. These transcription factors play roles in the lipogenesis of white adipocytes and thermogenic program of brown adipocytes (Inagaki, Sakai & Kajimura, 2016; Vernochet et al., 2009). However, another gene coordination is required for brown adipogenic differentiation (Kajimura, Seale & Spiegelman, 2010). A recent study on the same hMSC line, UE7T-13, showed that modifying the expressions of endogenous gene *PRDM16* along with other key regulators, such as *PPARγ*, *C/EBP-α*, and Kruppel-like factor 5, by CRISPR-Cas9 can direct these hMSCs toward WAT or BAT formation (Furuhata et al., 2017). In this study, we observed that BA did not affect the expression of *PPARγ* but decreased the expression of *C/EBP-α* along with the upregulation of *UCP-1* and *PGC-1*α. Induced small lipid droplets formation by BA, the key characteristic of brown adipocytes, was also observed in this work. The study in HFD mice also showed that BA improved thermogenic capacity in acute cold exposure test with increased in brown fat gene expressions including *UCP-1* and *PGC-1*α (Kim et al., 2019). *UCP-1* and *PGC-1*α regulate the mitochondrial biogenesis, oxidative metabolism, and thermogenesis of BAT (Park, Kim & Bae, 2014). The activation of *UCP-1* provides benefits against obesity, decreases fat mass, and improve insulin sensitivity (Li et al., 2000; Poher et al., 2015). Overall, our findings suggest that BA can help with balanced glucose uptake and lipid metabolism during induced adipogenesis in hMSCs.

## Conclusions

The present study examined the cellular responses of human mesenchymal stem cells, the progenitor cells of bone and fat tissues. The results suggested that betulinic acid increased osteogenesis and decreased adipogenesis in hMSCs. Decreases in leptin and adiponectin secretion were found at the secreted protein level but not at the mRNA expression. This indicated the potential control mechanisms of BA at the translation or cytokine releasing. Reduced size of lipid droplets along with the upregulation of UCP-1 and *PGC-1*α mRNA expressions suggested the potential effects of BA on brown adipogenic induction in human mesenchymal stem cells.

## Supplemental Information

10.7717/peerj.12321/supp-1 Supplemental Information 1 BA caused no alteration in protein content during hMSC adipogenesis at days 7, 10 and 14. Results are displayed as mean ± standard deviation (*n* = 3). *P < 0.05.

10.7717/peerj.12321/supp-2 Supplemental Information 2 GPDH activity of hMSCs grown in complete growth media (CGM) or adipogenic media (AM) at days 7 and 14. Results are displayed as mean ± standard deviation (*n* = 3). *P < 0.05.

10.7717/peerj.12321/supp-3 Supplemental Information 3 Microscopic images showed hMSC adipogenic differentiation at days 14 and 21. Phase-contrast (left panel) showed live-cells with lipid droplets appeared in clear round shape (arrows). Oil read O staining (right panel) confirmed the present of lipid droplets.

10.7717/peerj.12321/supp-4 Supplemental Information 4 Raw data for Metabolic activity.

10.7717/peerj.12321/supp-5 Supplemental Information 5 Raw data for osteogenesis and adipogenesis.

10.7717/peerj.12321/supp-6 Supplemental Information 6 Raw data for nile red staining.

10.7717/peerj.12321/supp-7 Supplemental Information 7 Raw data for glucose uptake.

10.7717/peerj.12321/supp-8 Supplemental Information 8 Raw data for cytokine secretion.

10.7717/peerj.12321/supp-9 Supplemental Information 9 Raw data for qRT-PCR.

## Additional Information and Declarations

### Data Availability

The following information was supplied regarding data availability: The raw data of each replicate for all results are available in the Supplemental Files.
