# Supplementary material for: Betulinic acid decreases lipid accumulation in adipogenesis-induced human mesenchymal stem cells with upregulation of PGC-1α and UCP-1 and post-transcriptional downregulation of adiponectin and leptin secretion
Source: PeerJ. 2021 Oct 14;9:e12321. doi: 10.7717/peerj.12321 (PMC8520689; doi:10.7717/peerj.12321)
Supplement: Supplemental Information 2 — Results are displayed as mean ± standard deviation (n = 3). *P < 0.05. [file peerj-09-12321-s002.docx]

**Supplemental** **Table S1:** GPDH activity of hMSCs grown in complete growth media (CGM) or adipogenic media (AM) at days 7 and 14. Results are displayed as mean ± standard deviation (n=3). **P* < 0.05.

| Time point | GPDH activity (mU/mg Protein) | |
| --- | --- | --- |
|  | CGM | AM |
| Day 7 | 21.51 ± 0.77 | 28.78 ± 3.02^*^ |
| Day 14 | 17.65 ± 1.39 | 20.69 ± 1.62 |
